# Knowledge-Driven Multi-Locus Analysis Reveals Gene-Gene Interactions Influencing HDL Cholesterol Level in Two Independent EMR-Linked Biobanks

**DOI:** 10.1371/journal.pone.0019586

**Published:** 2011-05-11

**Authors:** Stephen D. Turner, Richard L. Berg, James G. Linneman, Peggy L. Peissig, Dana C. Crawford, Joshua C. Denny, Dan M. Roden, Catherine A. McCarty, Marylyn D. Ritchie, Russell A. Wilke

**Affiliations:** 1 Department of Molecular Physiology and Biophysics, Center for Human Genetics Research, Vanderbilt University School of Medicine, Nashville, Tennessee, United States of America; 2 Biomedical Informatics Research Center, Marshfield Clinic Research Foundation, Marshfield, Wisconsin, United States of America; 3 Department of Biomedical Informatics, Vanderbilt University School of Medicine, Nashville, Tennessee, United States of America; 4 Division of Clinical Pharmacology, Department of Medicine, Vanderbilt University School of Medicine, Nashville, Tennessee, United States of America; 5 Department of Pharmacology, Vanderbilt University School of Medicine, Nashville, Tennessee, United States of America; 6 Center for Human Genetics, Marshfield Clinic Research Foundation, Marshfield, Wisconsin, United States of America; University of Swansea, United Kingdom

## Abstract

Genome-wide association studies (GWAS) are routinely being used to examine the genetic contribution to complex human traits, such as high-density lipoprotein cholesterol (HDL-C). Although HDL-C levels are highly heritable (h^2^∼0.7), the genetic determinants identified through GWAS contribute to a small fraction of the variance in this trait. Reasons for this discrepancy may include rare variants, structural variants, gene-environment (GxE) interactions, and gene-gene (GxG) interactions. Clinical practice-based biobanks now allow investigators to address these challenges by conducting GWAS in the context of comprehensive electronic medical records (EMRs). Here we apply an EMR-based phenotyping approach, within the context of routine care, to replicate several known associations between HDL-C and previously characterized genetic variants: *CETP* (rs3764261, p = 1.22e-25), *LIPC* (rs11855284, p = 3.92e-14), *LPL* (rs12678919, p = 1.99e-7), and the *APOA1/C3/A4/A5* locus (rs964184, p = 1.06e-5), all adjusted for age, gender, body mass index (BMI), and smoking status. By using a novel approach which censors data based on relevant co-morbidities and lipid modifying medications to construct a more rigorous HDL-C phenotype, we identified an association between HDL-C and *TRIB1*, a gene which previously resisted identification in studies with larger sample sizes. Through the application of additional analytical strategies incorporating biological knowledge, we further identified 11 significant GxG interaction models in our discovery cohort, 8 of which show evidence of replication in a second biobank cohort. The strongest predictive model included a pairwise interaction between *LPL* (which modulates the incorporation of triglyceride into HDL) and *ABCA1* (which modulates the incorporation of free cholesterol into HDL). These results demonstrate that gene-gene interactions modulate complex human traits, including HDL cholesterol.

## Introduction

To date, nearly 600 genome-wide association studies (GWAS) have been completed, investigating 150 distinct complex human traits [1], [2]. Circulating levels of high-density lipoprotein cholesterol (HDL-C), low-density lipoprotein cholesterol (LDL-C), and triglycerides (TG) are quantitative traits commonly measured in clinical practice and strongly associated with vascular disease, making them appealing traits to investigate from a statistical, clinical, and practical standpoint [3]. The genetic factors underlying variability in blood lipid levels have been extensively studied using GWAS, in cohorts of various design [4]–[16]. Through the construction of biobanks linked to electronic medical records (EMRs), the clinical community is now in a position to assess these associations in practice.

There is particularly strong interest in the characterization of genetic factors underlying population variability in HDL-C [17]. In human populations, every 1 mg/dl decrease in HDL-C is associated with a 6% increase in cardiovascular risk [18]. HDL particles also appear to have direct anti-atherogenic properties in animal models [19]. Therefore, while these smaller particles may serve as a source of cholesterol esters for the larger, more atherogenic LDL particles, the HDL particles themselves actually appear to attenuate the development of cardiovascular disease [20]. HDL is under tight genetic control (h^2^ up to ∼70%) [21], yet despite HDL's high heritability, even some of the most well powered GWAS studies have only explained a very small proportion of HDL variation using common SNPs [9], [12], [22]. This net unexplained variation due to genetics, often termed the “missing heritability” problem [23], has challenged GWAS studies for many complex traits beyond circulating lipid levels [2], [24].

A general explanation for this missing heritability is that it reflects forms of genetic variation that are not captured in the GWAS paradigm; these include rare genetic variation, structural variation [2], [23], epigenetics, and gene-gene (GxG) and gene-environment (GxE) interactions [2], [23], [25], [26]. GxG interaction (epistasis) is thought to be an important component of complex, multifactorial diseases due to the complexity of biological systems [27]. Data from animal models provide compelling support for the role of GxG interaction in the control of complex traits [28], [29]. Exploration of GxG in GWAS is often limited by lack of large sample sizes and statistical methods. One possible solution to the sample size problem is presented by the growing number of DNA repositories linked to electronic health records. These resources can provide cohorts of sufficient size for the characterization of GxG interaction. In parallel, computational capacity and novel methodologies have emerged to make the search for epistasis in GWAS feasible [30], [31].

Here we present data from a GWAS analyzing HDL-C using the Marshfield Clinic Personalized Medicine Research Project (PMRP) database [32], a node of the NHGRI-funded eMERGE network (*e*lectronic *M*edical *R*ecords and *Ge*nomics) [www.gwas.org]. We first conducted a genome-wide scan for SNPs associated with median HDL-C level using the secure encrypted comprehensive EMRs of 3947 PMRP participants. We also constructed a modeled HDL phenotype that accounts for environmental effects such as population trends in age, body mass index (BMI), and relevant co-morbidities [17]. We next investigated GxG interaction in this same dataset, using an approach that leverages existing biological knowledge [33]–[35].We required that any GxG interactions in this cohort (n = 3740 PMRP participants) showed evidence of replication in the de-identified EMRs of a second cohort from the eMERGE network (n  =  1858 records from the BioVU project [36]). This resulted in replicated GxG interactions associated with variation in HDL-C; all of which have potential biological relevance.

## Methods

### Ethics Statement

For the discovery cohort in the Marshfield PMRP, the study was approved by the Institutional Review Board of the Marshfield Clinic, and conducted in accordance with the basic principles of the Declaration of Helsinki. All study subjects in the discovery cohort provided written informed consent allowing access to their entire electronic medical record, through their participation in the Marshfield Clinic Personalized Medicine Research Project (PMRP). For our replication cohort we used samples chosen from BioVU, Vanderbilt's DNA databank. BioVU employs novel informatics strategies to de-identify Vanderbilt's comprehensive EMR and link these data to DNA extracted from blood samples obtained through routine clinical care and that would otherwise be discarded. Participants have the option of opting out at the clinical point of care, and the use of this de-identified research resource has therefore been determined by Vanderbilt's Institutional Review Board to represent non-human subject research. The federal Office of Human Research Protections concurred, and informed consent was therefore not required for access to the de-identified information within the BioVU biobank. We maintain a substantial oversight structure to promote the ethical maintenance of the resource, and to supervise all research conducted with its contents. Oversight bodies include: Vanderbilt University Institutional Review Board, the Vanderbilt University Medical Center Ethics Committee, the External Ethics Advisory Board, the Community Advisory Board, and the Vanderbilt University legal department [37]. The PMRP database and BioVU are both eMERGE nodes, and are two of the largest practice-based biobanks in the U.S. with over 20,000 in the PMRP and over 100,000 in BioVU as of December, 2010 [36], [38], [39].

### Phenotyping

To facilitate the construction of accurate prediction models for cardiometabolic risk, the eMERGE network has begun extracting clinical lipid data from multiple participating sites. Marshfield Clinic in Central Wisconsin has one of the oldest internally developed EMRs in the US, with coded diagnoses dating back to the early 1960's and laboratory observations dating back to 1985. The EMR data collected for clinical care is transferred daily into the Marshfield Clinic Data Warehouse where it is made available for research. We modeled lipid variables using this data source for the participants in the Marshfield PMRP cohort [17].

The PMRP demographics reflect the composition of the Central Wisconsin community, and the corresponding dataset has a distribution of fasting lipid levels similar to that reported by NHANES III [40]. At present, the PMRP Biobank contains data from over 20,000 adult participants; more than 10,000 individuals (54%) have impaired fasting glucose; >8,000 (41%) have hypertriglyceridemia; and >9,000 (48%) have reduced levels of HDL cholesterol according to criteria published by NCEP ATP-III [41], [42].

The eMERGE network design includes selection of ∼3,000 subjects with a predesignated phenotype at each node for genome-wide genotyping. In PRMP, the primary phenotype was cataract (n = 3,947 subjects). This phenotype resulted in a set enriched for older adults (age range 52 to 90 years, mean age 72). The set included 3,740 primarily European-American individuals with at least two fasting HDL measurements available for use in the present analysis. Due to the longitudinal nature of these data (2 to 78 lipid data points per individual; mean  =  14.4±10.1 data points), we defined two phenotypes to be used in the analyses below: (1) median HDL and (2) modeled HDL as previously described [17]. All HDL measurements for every individual were extracted from the EMR.

For the single-locus analysis of HDL level in the Marshfield PMRP, a simple median HDL level was computed for every individual who had at least two or more HDL datapoints in the record. Analyses using median HDL included adjustments for smoking, age, age^2^, BMI, BMI^2^, and gender, as all of these factors showed highly significant associations with median HDL. A second HDL measurement, termed here as modeled HDL, was also calculated for individuals in the Marshfield dataset. To calculate a modeled HDL for an individual, we extracted all lipid data and censored any HDL data acquired after lipid treatment or the onset of a relevant co-morbidity, and then used population-based trends in age and BMI to adjust individual estimates [17]. Records were censored at the first date of diagnosis for the following clinical co-morbidities known to influence circulating lipid levels: cancer, diabetes mellitus, and thyroid disease. Cancer (any malignancy except two common skin cancers - basal cell carcinoma and squamous cell carcinoma of the skin) was censored based on ICD-9 codes in the EMR. Thyroid disease and diabetes were assessed from the EMR using an electronic phenotyping algorithm available at the eMERGE website (www.gwas.org). Smoking status was acquired via information provided on a questionnaire provided at study entry, later confirmed by interview. Records were also censored at the first date of prescription for medications known to alter circulating lipid levels (either therapeutically or indirectly), including statins, fibric acid derivatives, niacin, and exogenous gonadal steroids. Our selection of these covariates has been published [17]. The data were extracted from the EMR using natural language processing (NLP) [43]–[46]. These algorithms have also been validated and published [43], and the programming pseudocode is freely available through the eMERGE network (www.gwas.org).

For the gene-gene interaction analysis, we replicated findings using independent samples from BioVU, the EMR-derived DNA databank at Vanderbilt University Medical Center [36], [47]. The primary eMERGE phenotype at this site is variability in the duration of the QRS complex on the normal electrocardiogram. Replication of the gene-gene interaction analysis in the Marshfield PMRP was conducted in 1,858 European-American and African-American individuals from the Vanderbilt BioVU dataset having at least two HDL measurements extracted from a de-identified synthetic derivative of the EMR. Because the additional clinical data mentioned above were not available for samples in this dataset, no modeled HDL could be computed on these data, and no adjustments were made in the gene-gene interaction analysis. It is, however, unlikely that any of these variables will confound any association to a gene-gene interaction replicating across both the Marshfield PMRP and Vanderbilt BioVU samples.

### Genotyping and Quality Control

Genotyping for the Marshfield PMRP samples was performed as part of the eMERGE network at the Center for Inherited Disease Research (CIDR) at Johns Hopkins University. The Illumina Human660W-Quadv1_A genotyping platform was used for this study. This platform consists of 560,635 SNPs and 96,731 intensity-only probes. Genotyping calls were made at CIDR using BeadStudio version 3.3.7. Our discovery cohort includes 3,947 samples from the Marshfield PMRP, 21 blind duplicates, and 85 HapMap controls. The HapMap controls include 44 CEPH, 32 Yoruba, 5 Japanese, and 4 Han Chinese; 40 independent HapMap replicate pair experiments, 19 independent HapMap trio experiments and 8 independent parent-child pairs were defined. The HapMap concordance rate was 99.8%. The blind duplicates reproducibility rate was 99.99%.

Genotyping for the Vanderbilt BioVU samples was performed as part of eMERGE primarily at the Broad Institute. A small number of BioVU samples, where the primary phenotype of interest was dementia were genotyped at CIDR. Similar to the PMRP samples, most samples were genotyped for SNPs on the Illumina Human660W-Quadv1_A platform, although a subset of these samples were genotyped on the Illumina 1M-Duo BeadChip (those who were known to be African American), which included all the SNPs on the 660W platform in addition to extra SNPs that were not considered in this analysis.

Genome-wide SNP data were cleaned using the eMERGE quality control (QC) pipeline developed by the eMERGE Genomics Working Group [48]. This process includes evaluation of sample and marker call rate, gender anomalies, duplicate and HapMap concordance, batch effects, Hardy-Weinberg equilibrium, Mendelian errors, sample relatedness, and population stratification. We also removed any SNPs with a minor allele frequency less than 1%, as power to detect associations with these variants was low. After QC and minor allele frequency filtering, 522,204 SNPs were used for the single locus analysis in the Marshfield PMRP dataset. Seven individuals were removed due to low (<99%) call rate. In the Marshfield PMRP cohort, over 98% of the participants self-reported European-American ancectry. Reconciling ancestry using self-report, principle components [49], and structured analysis resulted in exclsuion of a further 37 non-European genetic ancestry outliers. After excluding these samples, none of the top ten principal components were significant [49] or explained more than one tenth of one percent of HDL variation, and so no adjustment for principal components of ancestry was used in this sample. For the gene-gene interaction replication set using Vanderbilt BioVU samples in a combination of both European-Americans and African-Americans, we identified and excluded 5 ethnic outliers and adjusted analyses for two marginally significant principle components using Eigensoft [49]. All genotype data and detailed documentation of the quality control procedures summarized here have been deposited and are available at dbGaP [50].

### Statistical analysis

More than half of the individuals in the Marshfield dataset had a first, second, or third degree relative also in the dataset. To allow for the inclusion of related individuals without inflating the type I error rate in the single-locus analysis, we performed the GWAS analysis using a linear mixed effects analysis [51] with GenABEL [52] implemented in the R statistical computing environment [53]. Residuals from this model are essentially free from familial correlations, and can be used in a simple linear regression model for each SNP, which was performed using PLINK [54]. Genome-wide statistical significance was determined using a Bonferroni correction.

**Figure 1:**
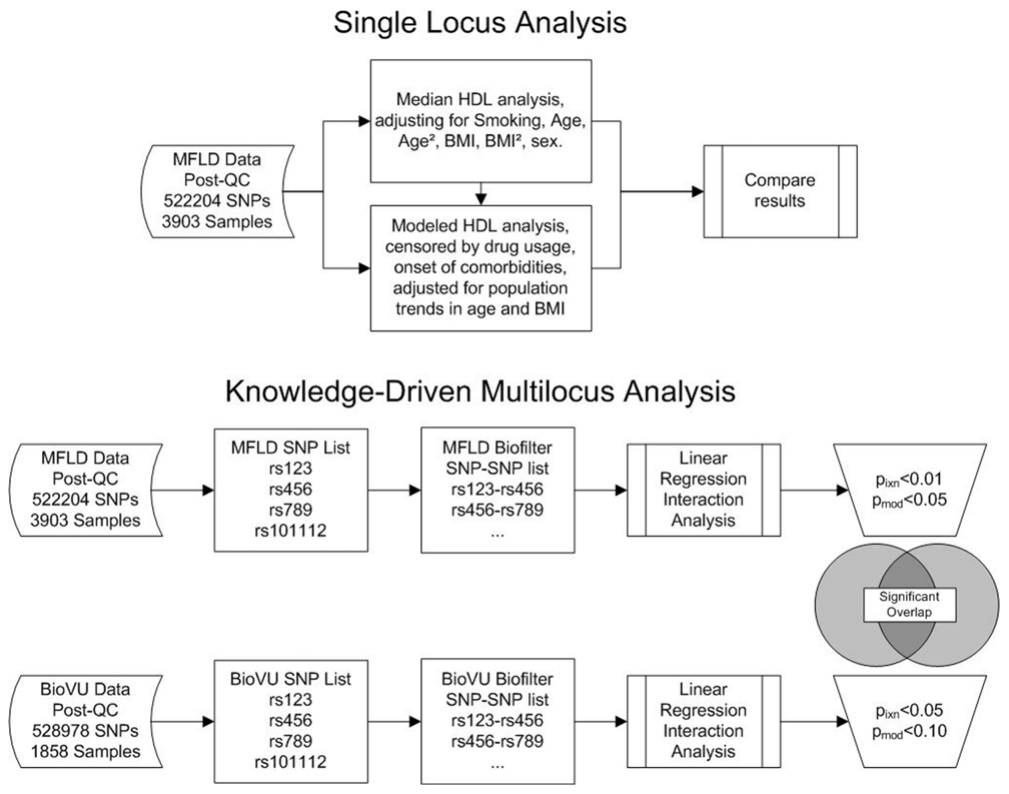
Flow diagram overview of the analysis plan. For the single locus analysis in the Marshfield PMRP cohort, a genome-wide association study was performed for both median and modeled HDL-C (see Phenotyping section in Methods). For the multilocus analysis, the Biofilter was used to generate putative multilocus interaction models that were tested for association with median HDL-C in the Marshfield PMRP cohort. The Vanderbilt BioVU cohort was used for replication. See the Methods and Results sections.

One frequently-cited causal mechanism underlying GxG interaction has been variability within multiple genes in similar pathways, protein families, or genes with similar or redundant biological function [33], [34]. For analyses of gene-gene interaction, the Biofilter is a bioinformatics algorithm that leverages domain knowledge from publicly available biological databases among SNPs from biologically plausible gene sets sharing physiological or biochemical similarity (or that have been previously associated with the phenotype under investigation) [35]. The rationale for using the Biofilter is to reduce both the computational and multiple testing burdens inherent in testing for gene-gene interactions. The Biofilter quantifies evidence in support of a particular gene-gene interaction model by counting the number of public database sources that independently link the two genes within a similar biological mechanism. Choosing to be conservative, we required that any gene-gene model be independently supported by four sources of biological knowledge among the following six: the Gene Ontology[55]; the Database of Interacting Proteins[56]; the Protein Families Database[57], [58]; the Kyoto Encyclopedia of Genes and Genomes (KEGG)[59]; Reactome[60]; or NetPath[61]. This criterion identified 22,769 SNP-SNP interactions that were tested for association to HDL-C levels in the Marshfield PMRP, and significant results were followed up using the Vanderbilt BioVU cohort. Figure 1 is a flow diagram illustrating an overview of the analysis plan.

## Results

### Sample Summary Statistics

Genome-wide association analyses were conducted in the Marshfield PMRP cohort using 522,204 SNPs which passed rigorous quality control procedures established by the eMERGE network. This analysis was performed for both median adjusted HDL-C and the modeled HDL-C phenotype (censored according to medication exposure and relevant co-morbidities). Table 1 summarizes the clinical characteristics of the Marshfield population. There were 1,541 male and 2,199 female study subjects (total 3,740) with clinical laboratory records containing at least two fasting HDL measurements that could be used in the median HDL-C analysis. Of these 3,740 samples, 2,190 had used statins, 883 have had hypothyroidism or hyperthyroidism, 733 have had cancer, 1,515 reported having ever smoked, and 301 are current smokers.

**Table 1:** Descriptive statistics of quantitative clinical variables in the Marshfield PMRP cohort.

	N	Median	Mean	SD	Min	Max
Age (years)	3,964	72	72.16	11.03	52	90
Weight (kilograms)	3,925	80.7	82.92	18.63	34.5	186
Height (centimeters)	3,927	165.1	166.88	9.63	121.9	195.6
BMI (kg/m^2^)	3,923	28.8	29.68	5.79	16.1	64.5
Modeled HDL-C (mg/dl)	2,528	49.1	50.8	13.44	19.8	114.7
Median HDL-C (mg/dl)	3,740	51	53.4	14.43	22	127.5

Nested within this group of 3,740 unique individuals, there were 1,142 male and 1,386 female study subjects (total 2,528) with a clinical laboratory record complete enough to construct a more rigorous modeled HDL-C phenotype (censored according to medication exposure and relevant co-morbidities). Although the sample size for modeled HDL-C was lower than the sample size for median adjusted HDL-C (because censoring left no analyzable data for some individuals), we performed the analyses using both phenotypes. Our reasoning was that modeled HDL-C has the potential of revealing novel associations otherwise masked by the presence of anabolic/catabolic disorders and/or clinical intervention.

Gender was the most reliable clinical predictor of median HDL-C level in the Marshfield PMRP cohort. The average HDL-C concentration (± standard deviation) was 45.9±10.9 mg/dl in males, and 58.5±14.3 mg/dl in females, p = 2e-163. This observation is consistent with the established literature [20], [42]. We therefore also included gender as a covariate in the model for each SNP association. The distribution of median HDL-C is shown in Figure S1. While slightly right-skewed, the distribution was approximately normal. We therefore fit a linear regression model for each SNP assuming an additive allelic model on untransformed HDL-C, as has been done previously [62]. To allow for related samples without inflating the type I error rate, regression on each SNP was performed using residuals from the linear mixed effects model that allowed for a random polygenic effect (see methods section).

### Single Locus GWAS Analysis: Median Adjusted HDL-C

**Figure 2:**
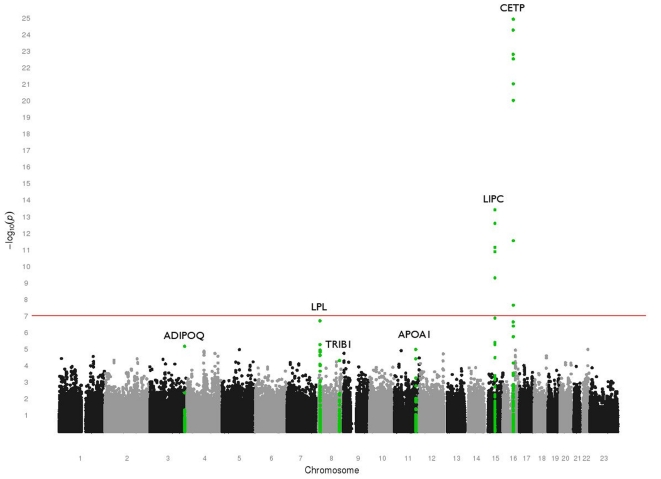
Summary of genome-wide association results. This plot shows the –log10(P-values) from linear mixed effects regression model using the natural log tranformed median HDL-C level in the Marshfield PMRP sample, adjusted for smoking, age, age^2^, BMI, BMI^2^, and gender. The red line indicates a Bonferroni-corrected significance threshold. Genes described in the results and the discussion sections are highlighted in green: *ADIPOQ* (chromosome 3), *LPL* (chromosome 8), *TRIB1* (chromosome 8), *APOA1/C3/A4/A5* (chromosome 11), *LIPC* (chromosome 15), and *CETP* (chromosome 16).

Figure 2 summarizes the results from our genome-wide association study, showing the –log10(P-value) for each SNP from the analysis of median HDL-C level, adjusted for smoking, age, age^2^, BMI, BMI^2^, and gender. Figure S2 shows the quantile-quantile plot of the –log10(P-values) from this analysis plotted against the expected distribution of P-values under the null hypothesis. Median adjusted HDL-C level in the Marshfield PMRP cohort was very strongly associated with SNPs in cholesterol ester transfer protein (*CETP*) on chromosome 16, and strongly associated with hepatic lipase (*LIPC*) on chromosome 15. Both *CETP* and *LIPC* have previously been implicated in cholesterol homeostasis in other genome-wide association studies [4], [9], [10], [12], [16]. The SNP with the strongest evidence for association with median HDL-C was rs3764261 (p = 1.22e-25), 2.5kb upstream of the *CETP* transcription start site. This SNP alone accounted for approximately 3% of the variance in median adjusted HDL-C level. This SNP is in LD (r^2^>0.8 in HapMap CEU) with rs247616 (not genotyped here), an eQTL which has been demonstrated to contribute to cis-regulation of *CETP* mRNA levels in HapMap lymphoblastoid cell lines [63]. There were several other strongly associated variants upstream of the *CETP* transcription start site, and we observed association between HDL-C and a well-characterized nonsynonymous coding SNP, rs5882 (p = 4.1e-7), known to encode a valine to isoleucine change at amino acid position 422 in the *CETP* gene product. A previous study in Ashkenazi Jews with exceptional longevity has revealed that individuals with the V/V genotype at this site demonstrate increased lipoprotein sizes and lower serum *CETP* concentration, both of which are heritable and promote successful aging [64].

The other signal observed at a level of genome-wide significance was in hepatic lipase (*LIPC*). Variants in this gene have also been associated with HDL-C level in several previous studies [4], [9], [10], [12], [16]. The SNP in the *LIPC* locus with the strongest association in our study cohort was rs11855284 (p = 3.92e-14), residing 13.5kb upstream of the *LIPC* transcription start site on chromosome 15. The most significantly associated SNP within the *LIPC* genic region was intronic SNP rs261336 (p = 5.25e-6). These two SNPs explained 1.6% and 0.5% of the variance in HDL-C cholesterol, respectively.

Our most significant signal that did not meet genome-wide significance was rs12678919 (p = 1.99e-7), a SNP in an intergenic region 19kb downstream of lipoprotein lipase (*LPL*) on chromosome 8. This SNP explained 0.7% of the variance in HDL cholesterol. It is noteworthy that a different variant at the *LPL* locus (rs253) was identified in subsequent gene-gene interaction analyses here. Details are discussed further below.

### Single Locus GWAS Analysis: Modeled HDL-C

**Figure 3:**
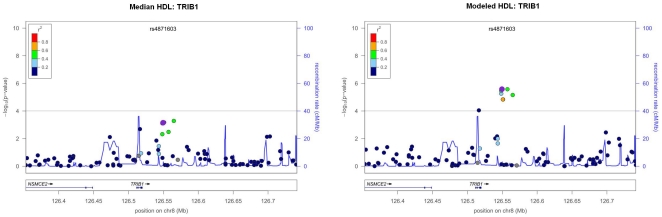
Association results for the TRIB1 region. This shows the association results for the Tribbles 1 Homolog (*TRIB1*) region, for median adjusted HDL-C (left) and modeled HDL-C (right) in the Marshfield PMRP cohort. Color scale displays the degree of linkage disequilibrium (r^2^) between markers. Blue line shows recombination rate from HapMap CEU. Gene location is shown along the horizontal axis of each panel. This plot shows that while the statistical significance of the effect of *TRIB1* on HDL-C levels is less compelling when adjusting for triglyceride concentration, the association is much stronger when using the modeled HDL-C phenotype (even though this phenotype has far fewer samples than the median adjusted HDL-C phenotype). SNP rs4871603 in *TRIB1* was associated with median HDL-C at p = 7.06e-4 and with modeled HDL-C at p = 2.61e-6 without adjusting for triglyceride concentration. After adjusting for triglycerides, the p-values for median and modeled HDL-C become less significant (p = 0.296 and p = 0.0056, respectively, data not displayed).

Our data were derived from an electronic medical record. Therefore, we also tested each SNP for genome-wide association with modeled HDL-C as the outcome, a trait which censors by onset of relevant co-morbidities or usage of lipid-modifying drugs and adjusts for population trends in age and BMI [17]. Because this more rigorous phenotype required data for several covariates, complete data was only available on 2,528 samples in the Marshfield PMRP cohort. Using 1,156 fewer samples we still reproduced the genome-wide significant association to *CETP* (rs3764261, p = 2.63e-13), and a highly significant association to both *LPL* (rs1441762, p = 1.53e-6) and *LIPC* (rs11856159, p = 1.59e-6). Full results are available via Table S1 for SNPs associated at p<1×10^-5^ with either median or modeled HDL-C. Interestingly, even though using modeled HDL-C as the phenotype resulted in a decreased sample size, the association signal was stronger for certain regions of the genome than when using median adjusted HDL-C. Despite using lower numbers, we observed improvement in the strength of association by more than two orders of magnitude, for many variants included in this genome-wide SNP scan, when the HDL-C trait was modeled (e.g., *CENTG2*, *COL23A1*, *CACNA2D1*, *CYB5B*, *FHOD3*, *GBX2*, *TRIB1*, *TLE4*, *TMEM135*, *UBE3A*). As shown in Figure 3, this effect was most pronounced for SNPs in or near *TRIB1*, the Tribbles homolog 1 gene (rs2385114, an intronic SNP, p = 8.96e-5; rs4871603, 30kb downstream, p = 2.61e-6). Our ability to resolve this association strengthened when these variables were considered. *TRIB1* was also associated at the genome-wide level with triglyceride concentration in our data (most significant SNP, rs6982502, p = 3.7e-9, data not shown). Because we observed a very strong logarithmic correlation between median triglyceride level and median HDL-C cholesterol level (Figure S3), we further assessed the *TRIB1*-HDL-C relationship using multiple linear regression, adjusting for triglyceride concentration in addition to the other clinical variables (e.g., age, gender, BMI and smoking status). In the analysis adjusting for triglyceride level the effect of *TRIB1* was highly attenuated (p = 0.0056). These results emphasize the need for evaulating data with and without strategies that censor the longitudinal strings of lipid data based on medication (e.g., niacin) and relevant co-morbidity (e.g., diabetes).

### Gene-Gene Interaction Analysis and Replication

We have demonstrated that biobanks linked to EMR data can provide a unique resource for robust replication of previous GWAS findings, and they have the capability of uncovering novel genetic associations. However, as in other studies, even our most significant findings individually explain only a small proportion of the variance in HDL-C level. We next leverage both the PMRP cohort and a similarly phenotyped cohort from the Vanderbilt BioVU EMR-linked biobank to investigate gene-gene interactions and HDL-C. Table 2 summarizes the clinical characteristics of the Vanderbilt BioVU population.

**Table 2:** Descriptive statistics of quantitative clinical variables in the Vanderbilt BioVU cohort.

	N	Median	Mean	SD	Min	Max
Age (years)	2,576	57	56.56	15.85	19	90
Weight (kilograms)	2,522	85.16	88.05	24.62	36.29	251.74
Height (centimeters)	2,294	167.64	168.5	10.01	129.54	203.2
BMI (kg/m^2^)	2,292	29.44	30.98	7.94	14.63	72.5
Median HDL-C (mg/dl)	2,576	50.0	53.20	17.320	9.0	146.0

The number of possible SNP-SNP interactions among 522,204 SNPs is over 1.36×10^11^. In addition to being extremely computationally intensive, exhaustive evaluation of all possible pairwise (SNP-SNP) interactions among GWAS data comes with an extraordinary loss of power due to the extremely large number of statistical tests being performed. This mandates prioritization of which interactions to test based on some intrinsic aspect of the data or other extrinsic domain knowledge. One data-driven approach is to select SNPs based on the strength and statistical significance of their independent main effects, evaluating interactions only between SNPs that meet a certain effect size or significance threshold [65]. This strategy makes the simplifying but unnecessary assumption that gene-gene interactions affecting the phenotype can only occur between SNPs that independently have a detectable effect on the trait. Instead, we used the Biofilter (described in the methods section) to prioritize a small subset of SNP-SNP interactions to test for association with HDL-C level using existing extrinsic biological knowledge. It is of note that this method does not require that any SNP have an independent statistically significant main effect.

We performed a gene-gene interaction analysis using multiple linear regression allowing for a multiplicative interaction term between the two additive-encoded SNPs. Requiring that SNP-SNP models be supported by at least four sources of extrinsic domain knowledge (see *Statistical Analysis* in Methods section), we tested 22,769 SNP-SNP interactions in the Marshfield PMRP cohort. Two test statistics were generated for each model tested: a t-test on the multiplicative interaction term (P_ixn_), and an F-test on the overall model (P_mod_). A significant P_ixn_ indicates a significant non-zero multiplicative gene-gene interaction between the two SNPs while a significant P_mod_ indicates a significant overall model (i.e. R^2^>0). We required both statistics to be significant in both datasets, further constraining our results to well-fitting models with strong evidence of non-additive gene-gene interaction.

Using this approach, we found 11 models with a significant interaction term and ANOVA p-values (P_ixn_<0.01 and P_mod_<0.05), indicative of non-additive gene-gene interaction in a well-fitting model contributing to median HDL-C level. Because of the linkage disequilibrium existing between SNPs in the same gene, and because tests of SNP-SNP interaction often include the same SNP being tested for interaction with other SNPs, the test statistics from this procedure can be highly correlated. Furthermore, an appropriate permutation test would require permutation of not only the phenotype but permutation of the SNP-SNP linkages in our sources of biological knowledge, and methodology to appropriately carry out such a permutation procedure has yet to be developed or tested. Because it is unclear how to properly correct for multiple testing in these datasets, we opted to require replication in a second cohort. These 11 SNP-SNP interaction models were therefore tested in the BioVU cohort, adjusting for two significant principal components to avoid any potential confounding by population stratification. Of the 11 models that were significant in the initial screen, six replicated in the BioVU replication cohort. Statistical significance thresholds for the replication cohort were slightly more liberal (P_ixn_<0.05, P_mod_<0.1) to avoid excessive type II errors due to the smaller sample size.

The results highlighting these 11 significant gene-gene interaction models are summarized in Table 3 (models indicated by one or more stars). The six models that show evidence for replication in the BioVU cohort are indicated by two or more stars. These six models are representative of four distinct gene-gene interactions: *GALNT1-GALNT2*, *GALNT2-GALNT3* (members of the GalNAc-transferases family), *LPL-ABCA1* (lipoprotein lipase and ATP Binding Casette A1), and *RPA2-RPA3* (Replication Protein A 2/3).

**Table 3:** Gene-gene interaction models.

REP	SNP 1	Gene 1	SNP 2	Gene 2	M β_1_	M β_2_	M β_3_	M P_ixn_	M P_mod_	M R^2^	V β_1_	V β_2_	V β_3_	V P_ixn_	V P_mod_	V R^2^
*	rs3927911	*BCL2*	rs4645900	*BAX*	0.213	3.901	−3.890	0.004	0.018	0.003	0.805	5.397	−5.808	0.042	0.154	0.003
*	rs2271709	*C7*	rs6699859	*C8A*	1.203	1.068	−1.776	0.005	0.028	0.002	−1.173	−1.176	2.433	0.020	0.138	0.003
*	rs910497	*GALNT2*	rs4621175	*GALNT3*	−0.727	−1.250	2.347	0.003	0.013	0.003	−0.890	−1.976	2.148	0.024	0.129	0.003
*	rs4621175	*GALNT3*	rs4846930	*GALNT2*	−1.213	−0.726	2.291	0.004	0.014	0.003	−1.750	−0.955	2.261	0.017	0.100	0.003
*	rs4621175	*GALNT3*	rs10864732	*GALNT2*	−1.179	−0.726	2.243	0.004	0.017	0.003	−1.641	−0.985	2.245	0.019	0.106	0.003
**	rs886724	*RPA3*	rs7536088	*RPA2*	1.493	1.713	−1.818	0.000	0.002	0.004	−2.064	−1.266	1.995	0.019	0.099	0.003
**	rs886724	*RPA3*	rs17257252	*RPA2*	0.890	1.182	−1.703	0.003	0.029	0.002	−2.035	−1.938	2.795	0.007	0.046	0.004
**	rs901675	*GALNT2*	rs4621175	*GALNT3*	1.216	2.109	−2.521	0.004	0.004	0.004	−2.114	−1.512	2.535	0.037	0.077	0.004
**	rs1471915	*GALNT2*	rs12963790	*GALNT1*	−0.410	−0.447	2.778	0.004	0.020	0.003	−2.114	0.098	−3.487	0.037	0.002	0.008
***	rs253	*LPL*	rs2515614	*ABCA1*	−0.340	−1.098	1.441	0.006	0.011	0.003	−0.618	−2.797	2.790	0.001	0.006	0.007
***	rs253	*LPL*	rs2472509	*ABCA1*	−0.338	-1.113	1.438	0.006	0.011	0.003	−0.399	−2.797	2.790	0.001	0.006	0.007

This table shows 11 significant gene-gene interaction models discovered in the Marshfield PMRP cohort (M P_ixn_<0.01 and M P_mod_<0.05, indicated by one to three stars). Six of these models show evidence for replication in the Vanderbilt BioVU cohort (M P_ixn_<0.05, M P_mod_<0.1, indicated by two to three stars). In two of these replicating models, all three pairs of coefficients were in the same direction in both datasets (indicated by three stars). The table shows the two SNPs and their corresponding genes involved in the gene-gene interaction. All SNPs here were intronic SNPs. The coefficients for the main effects (β_1_ and β_2_) and the interaction term (β_3_) are shown for both the Marshfield cohort (prefixed by “M”), and the Vanderbilt cohort (prefixed by “V”). Also shown are the interaction term p-values (P_ixn_), ANOVA model fit p-values (P_mod_), and overall R^2^ statistics for both the Marshfield and Vanderbilt cohorts (prefixed by “M” and “V” respectively).

We then refined our results highlighting models that were significant in the Marshfield cohort, replicate in the BioVU cohort, and where all coefficients were in the same direction – that is, if the coeffieicnt for either SNP or the interaction term is positive in the Marshfield cohort, the corresponding coefficient must also be positive in the BioVU dataset. This stringent criterion further reduced our replicating models to two similar interaction models involving *LPL* and *ABCA1* (models indicated by three stars in Table 3): rs253×rs2515614 and rs253×rs2472509. These two models were statistically redundant - the *ABCA1* SNPs (rs2515614 and rs2472509) were in extremely high linkage disequilibrium (r^2^ = 1 in Marshfield, r^2^ = 0.99 in BioVU), resulting in nearly identical coefficients and test statistics. One characteristic of this *LPL-ABCA1* interaction warrants special emphasis. In this model the direction of the main effects (β_1_ and β_2_) were all in the same direction, while the interaction effects were in the opposite direction. That is, in both datasets, inheriting a minor allele at either locus (but not both) results in a dosage-dependent decrease in HDL-C level, while inheriting a minor allele at both variants results in a change that is significantly higher than the expected change caused by the additive effects of both variants alone. It may be that, while either SNP alone can impact the production and/or vascular remodeling of HDL particles, the two variants together alter the TG/HDL ratio and particle stability.

## Discussion

We performed a genome-wide association analysis of HDL cholesterol level in two large clinical practice-based biobanks. We observed a number of previously reported associations between HDL-C level and genes impacting lipoprotein homeostasis (e.g., *CETP*, *LIPC*, *LPL*). Furthermore, our approach using an electronic phenotyping algorithm to censor based on clinical factors known to influence HDL-C cholesterol levels allowed us to identify genes that are more strongly associated in the context of clinical covariates (e.g.,*TRIB1*). Finally, we investigated gene-gene interaction using a novel bioinformatics approach that restricts the number of pairwise tests based on existing biological knowledge.

The primary phenotype utilized in this study was median HDL cholesterol level, derived from longitudinal clinical data. This trait was determined from laboratory data obtained during routine clinical care, and available within each subject's individual electronic medical record. In the single-locus analysis, the SNP most strongly associated with median HDL-C level was rs3764261 (p = 1.22e-25), 2.5 kb upstream of the *CETP* transcription start site on chromosome 16. This SNP alone accounted for approximately 3% of the variance in median adjusted HDL-C level. This same variant had also been reported in another genome-wide association study in the Northern Finland Birth Cohort (p = 6.97e-29) [12], and a meta analysis of three genome-wide association studies initially comprising 8,656 individuals and ∼2,261,000 imputed and/or genotyped SNPs (p = 2.8e-19) [16], followed by validation in six European cohorts totaling 11,569 individuals (p = 6.4e-43) [16]. Other studies have found different SNPs in the region upstream of *CETP* to be even more highly associated with HDL cholesterol levels: rs1800775 (p = 1e-73) in [9], rs173539 (p = 4e-75) in [10] where samples were combined from the two previously cited studies, and rs1532624 (p = 9.4e-94) in [4]. The SNP with the strongest association in our dataset (rs3764261) is in LD with an eQTL SNP which affects mRNA levels of *CETP* in HapMap lymphoblastoid cell lines [63]. The *CETP* gene product has known biological relevance, redistributing cholesterol esters and triglycerides between HDL-C particles and the larger, more atherogenic lipoproteins.

Free fatty acids and triglycerides are liberated from HDL-C particles through the activity of three well-characterized lipolytic enzymes (*LIPC*, *LIPG*, and *LPL*). In our dataset, a SNP in the first gene reaching genome-wide significance for association with HDL-C level was rs11855284 (p = 3.90e-14), 13.5kb upstream of the hepatic lipase (*LIPC*) transcription start site on chromosome 15. Further, our most significant signal that did not meet genome-wide significance was rs12678919 (p = 1.99e-7), a SNP in an intergenic region 19kb downstream of lipoprotein lipase (*LPL*) on chromosome 8. Thus, we observed association with *LPL* and *LIPC* but not *LIPG*. These observations are consistent with existing biological knowledge. The enzymatic activity of LPL favors lipolysis of triglyceride (i.e., phospholipase activity is relatively minor). Triglyceride rich HDL-C particles are less stable, and are quickly shuttled back to the liver for elimination, as SRB1 mediated removal is a function of triglyceride enrichment in HDL-C particles. Conversely, *LIPG* has relatively little TG-lipase activity, (i.e., primarily a phospholipase), and any common variants that affect expression or function of *LIPG* would have small effect on HDL-C stability, requiring a very large sample size to detect an association (∼9,800 samples to achieve 80% power to detect an effect of similar magnitude as has been previously observed for *LIPG* [16] at p = 1e-7).

When we used our previously developed approach to modeling HDL-C within an electronic record, which censors data based on relevant co-morbidities (e.g., diabetes mellitus) and lipid modifying medications (e.g., niacin), we identified several loci that were more strongly associated, with HDL-C, even though we had a reduced sample size. SNPs in and near *TRIB1* were more strongly associated with modeled HDL-C level using a lower sample size (n = 2528, p = 2.61e-6), versus using a simple median HDL-C (n = 3903, p = 7.06e-4). *TRIB1* resisted identification in all previous GWAS studies of HDL-C, and was only recently reported for the first time in a meta-analysis of over 100,000 samples[62]. In our data, HDL-C and triglyceride levels were logarithmically correlated (Figure S3), and the association between rs4871603 in *TRIB1* and modeled HDL-C (p = 2.61e-6) was markedly reduced after adjusting for triglyceride level (p = 0.0056). Further study is warranted to fully characterize the biology underlying this interaction. *TRIB1* may serve as a clinical surrogate for the triglyceride effect on HDL-C concentration. *TRIB1* encodes a G-protein-coupled-receptor induced protein involved in the function of mitogen-activated protein kinases [66], and the role of HDL-C in reverse cholesterol transport may be modulated through such an interaction [67]−[69]. Because *TRIB1* has previously been associated with coronary artery disease [4], [9], [10], [16], the *TRIB1*-HDL-C relationship observed within our data may have a profound impact on public health.

Our most significant findings, however, still explain less than 5% of the variance in HDL-C level, a trait that is up to 70% heritable. We therefore examined a small subset of all the possible gene-gene interactions in our GWAS data. We found 11 interactions that were nominally significant in our discovery cohort (the Marshfield PMRP biobank). After evaluating these models in a validation sample (Vanderbilt BioVU cohort), we found that six of the 11 models replicated. We required both the p-value on the interaction term and the p-value on the ANOVA F-test for the regression model to be significant in both datasets, further constraining our results to models with evidence of nonlinear gene-gene interaction in a well-fitting model. These models individually explained 0.2−0.8% of the variation in HDL-C in the Marshfield and BioVU cohorts (see Table 3). Only one set of observed gene-gene interactions showed evidence of replication in both datasets with consistent directionality in all three coefficients: *LPL* and *ABCA1*. Because we required evidence of replication with consistent direction of effect in a second dataset, we did not require a stringent multiple testing correction. While it would be possible to randomize the outcome data and permute this entire procedure, this method of permutation testing would be computationally intensive, and such a method has not yet been standardized for the permutation of the SNP-SNP linkages available in our sources of biological knowledge. Furthermore, this permutation test on an interaction term would have a more restrictive null hypothesis (no association) than the null hypothesis we wish to test (no interaction). Biologically, *LPL* mediates the release of free fatty acids and triglyceride from HDL-C particles, while *ABCA1* moves free cholesterol into HDL-C particles as they undergo intravascular remodeling. Thus, it is not surprising that we observed a statistically meaningful interaction between variants in these two genes. It is however surprising that the direction of the main effect coefficients (β_1_ and β_2_) were in the same direction while the interaction effect was in the opposite direction. While still considered a nonlinear epistatic interaction, the structure of the model in Table 3 is referred to as a *heterogeneity model* [70], [71] rather than a synergistic multiplicative model. In a separate study of type I diabetes, investigators found that four out of five statistically significant gene-gene interactions were also of this type [72]. Genetic heterogeneity is a serious concern with large-scale genetic studies, and is often cited as a reason for the widespread lack of replication in GWAS studies [73], [74]. Others have recently argued that epistatic genetic heterogeneity should be considered when analyzing genetic data for association to complex human traits [75]. Despite the fact that statistical tools, such as random forests, have been available for some time now to accomplish this [76], [77], analyses of GWAS data accounting for the possibility of epistatic heterogeneity is a task rarely undertaken. Accounting for genetic heterogeneity in genetic studies of complex disease may improve the replicability of findings in genome-wide studies of lipid and other phenotypes.

In summary, we have used EMR data from genotyped biobanked samples to perform the first knowledge-driven gene-gene interaction analysis for HDL-C. Using a second EMR-linked biobank cohort we demonstrated evidence for replication of a gene-gene interaction between *LPL* and *ABCA1*. As demonstrated here and elsewhere [36], [78], biobank-linked EMRs provide an excellent resource for genetic studies of complex traits. By utilizing the EMR to construct a rigorous phenotype and by accounting for gene-gene interaction as presented here, perhaps more variation can be explained and new biology discovered in complex traits like HDL-C level.

## Supporting Information

Figure S1 Distribution of HDL-C concentration (mg/dL) in males (blue) and females (red) in the Marshfield PMRP dataset.
(TIF)

Figure S2 Quantile-quantile plot of the –log10(P-values) from the median adjusted HDL-C analysis in the Marshfield PMRP cohort plotted against the expected null distribution.
(TIF)

Figure S3 Median HDL-C and median triglyceride concentrations are highly logarithmically correlated (r^2^ = .258). Trend line ± 95% confidence interval is shown.
(TIF)

Table S1 All single locus SNPs associated at p<1×10-5 with either median HDL-C or modeled HDL-C in the Marshfield cohort.
(PDF)
