# Supplementary figures and images for: Knowledge-Driven Multi-Locus Analysis Reveals Gene-Gene Interactions Influencing HDL Cholesterol Level in Two Independent EMR-Linked Biobanks
Source: PLoS One. 2011 May 11;6(5):e19586. doi: 10.1371/journal.pone.0019586 (PMC3092760; doi:10.1371/journal.pone.0019586)

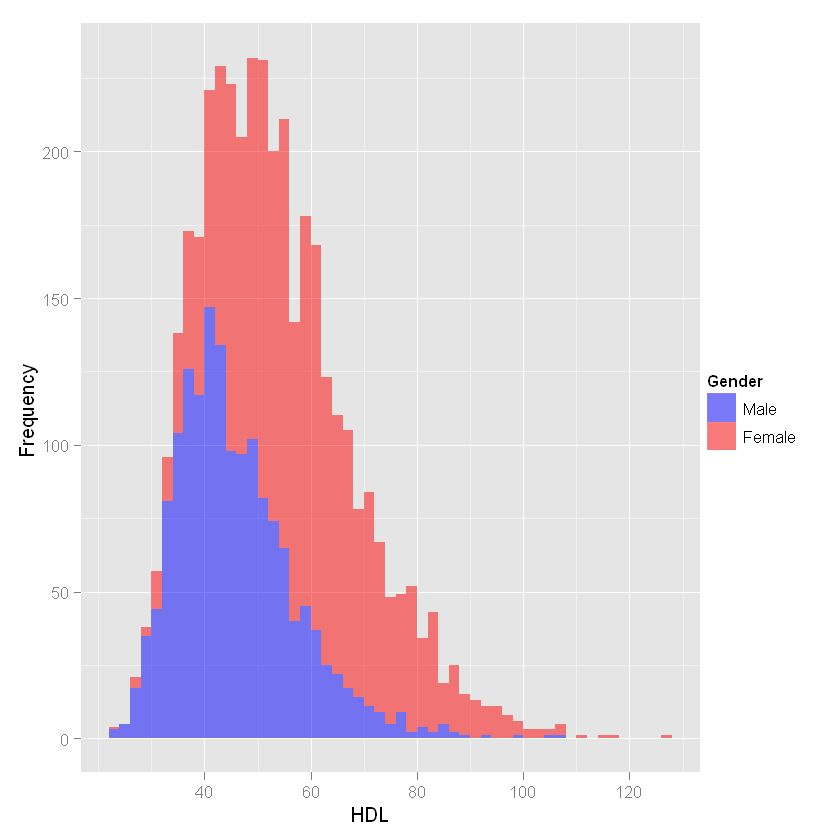

Supplement: Figure S1 — Distribution of HDL-C concentration (mg/dL) in males (blue) and females (red) in the Marshfield PMRP dataset. (TIF) [file pone.0019586.s001.tif]

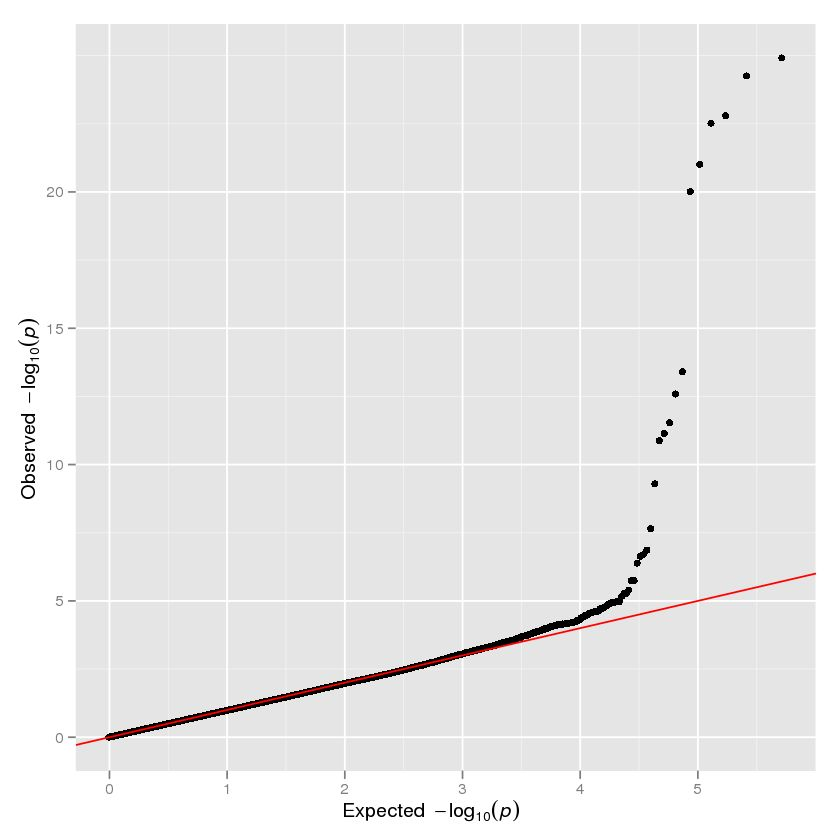

Supplement: Figure S2 — Quantile-quantile plot of the –log10(P-values) from the median adjusted HDL-C analysis in the Marshfield PMRP cohort plotted against the expected null distribution. (TIF) [file pone.0019586.s002.tif]

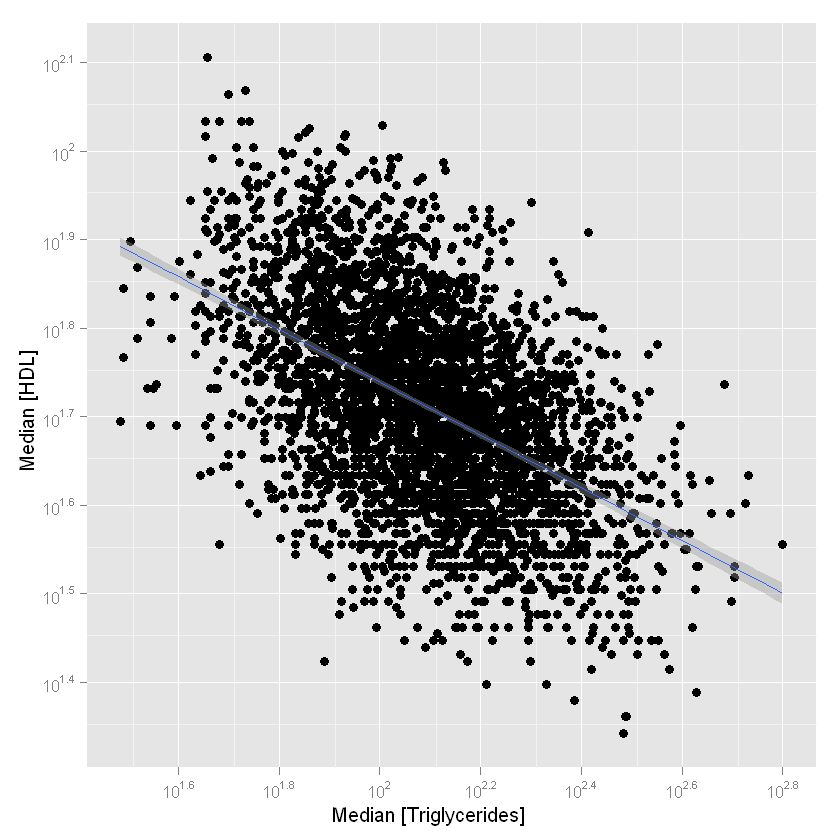

Supplement: Figure S3 — Median HDL-C and median triglyceride concentrations are highly logarithmically correlated (r2 = .258). Trend line ± 95% confidence interval is shown. (TIF) [file pone.0019586.s003.tif]
