# Supplementary material for: Knowledge-Driven Multi-Locus Analysis Reveals Gene-Gene Interactions Influencing HDL Cholesterol Level in Two Independent EMR-Linked Biobanks
Source: PLoS One. 2011 May 11;6(5):e19586. doi: 10.1371/journal.pone.0019586 (PMC3092760; doi:10.1371/journal.pone.0019586)
Supplement: Table S1 — All single locus SNPs associated at p<1×10^-5^ with either median HDL-C or modeled HDL-C in the Marshfield cohort. (PDF) [file pone.0019586.s004.pdf]

Supplementary Table 1. All single locus SNPs associated at $p<1\times10^{-5}$ with either median HDL-C or modeled HDL-C in the Marshfield cohort.

Chromosome	Position	SNP	Genes (w/in 50kb)	β Median	β Modeled	SE Median	SE Modeled	*T* Median	*T* Modeled	*P* Median	*P* modeled
3	188116469	rs7627293	*ST6GAL1*	2.288	1.116	0.508	0.6931	4.504	1.61	6.89E-06	0.1076
8	19860651	rs297	*LPL*	1.039	1.499	0.2399	0.3309	4.332	4.53	1.52E-05	6.16E-06
8	19864685	rs331	*LPL*	1.022	1.534	0.233	0.3208	4.388	4.784	1.18E-05	1.82E-06
8	19875201	rs10096633	*LPL*	1.359	1.748	0.2985	0.4053	4.554	4.312	5.42E-06	1.68E-05
8	19888502	rs12678919	*LPL*	1.752	1.987	0.3362	0.4594	5.211	4.325	1.99E-07	1.58E-05
8	19908967	rs1441762	*LPL*	0.9856	1.543	0.2327	0.3202	4.236	4.82	2.34E-05	1.53E-06
8	19909455	rs2083637	*LPL*	0.9832	1.5	0.2327	0.3203	4.225	4.682	2.45E-05	2.99E-06
8	126548497	rs6982636	*TRIB1*	0.57	1.3	0.2018	0.2791	2.825	4.657	0.004759	3.38E-06
8	126548544	rs6982502	*TRIB1*	-0.6817	-1.283	0.2028	0.2817	-3.361	-4.553	0.0007848	5.53E-06
8	126549549	rs4871603	*TRIB1*	-0.7327	-1.395	0.2161	0.2961	-3.39	-4.71	0.0007058	2.61E-06
8	126557432	rs2980869	*TRIB1*	0.5925	1.31	0.2014	0.2784	2.941	4.707	0.003289	2.66E-06
8	126565000	rs10808546	*TRIB1*	0.7002	1.25	0.2017	0.2773	3.472	4.505	0.0005225	6.93E-06
11	116154127	rs964184	*APOA1/C3/A4/A5*	-1.333	-2.019	0.3023	0.4087	-4.41	-4.939	1.06E-05	8.37E-07
15	56465804	rs10468017	*LIPC*	1.646	1.405	0.2241	0.3052	7.343	4.603	2.57E-13	4.38E-06
15	56470658	rs1532085	*LIPC*	1.432	1.078	0.2083	0.2849	6.874	3.784	7.30E-12	0.0001582
15	56476479	rs11855284	*LIPC*	1.997	1.771	0.263	0.3579	7.594	4.948	3.92E-14	8.02E-07
15	56478046	rs415799	*LIPC*	-1.075	-0.7406	0.2037	0.2807	-5.28	-2.638	1.36E-07	0.00838
15	56481152	rs487766	*LIPC*	1.543	1.249	0.2476	0.3393	6.234	3.682	5.08E-10	0.0002362
15	56486302	rs11856159	*LIPC*	1.752	1.682	0.2582	0.3497	6.786	4.811	1.34E-11	1.59E-06
15	56510771	rs1077834	*LIPC*	1.139	0.9811	0.2466	0.3406	4.617	2.881	4.03E-06	0.004001
15	56529710	rs261336	*LIPC*	1.253	1.189	0.2748	0.376	4.561	3.162	5.25E-06	0.001584
16	55542640	rs9989419	*CETP*	-1.442	-1.449	0.2058	0.28	-7.007	-5.175	2.89E-12	2.47E-07
16	55550825	rs3764261	*CETP*	2.25	2.138	0.2133	0.2907	10.55	7.352	1.22E-25	2.63E-13
16	55552395	rs4783961	*CETP*	0.9799	0.8336	0.2049	0.2789	4.782	2.989	1.81E-06	0.002826
16	55552737	rs1800775	*CETP*	2.068	1.928	0.1989	0.2698	10.4	7.146	5.55E-25	1.17E-12
16	55553789	rs708272	*CETP*	1.955	1.943	0.2028	0.2763	9.64	7.033	9.79E-22	2.60E-12
16	55554734	rs1864163	*CETP*	-2.161	-1.832	0.23	0.3156	-9.395	-5.806	9.78E-21	7.19E-09
16	55560647	rs11076174	*CETP*	-1.708	-1.406	0.3571	0.4834	-4.782	-2.909	1.81E-06	0.003661
16	55562980	rs1532624	*CETP*	2.015	2.006	0.2015	0.274	10	7.32	3.00E-23	3.33E-13
16	55564091	rs7499892	*CETP*	-2.654	-2.412	0.2637	0.361	-10.06	-6.68	1.61E-23	2.94E-11
16	55568686	rs4784744	*CETP*	-1.119	-1.025	0.216	0.295	-5.182	-3.473	2.32E-07	0.0005234
16	55573593	rs5882	*CETP*	1.117	0.9384	0.2202	0.3048	5.073	3.079	4.10E-07	0.002103
16	55575603	rs289744	*CETP*	1.247	1.104	0.2226	0.3075	5.603	3.591	2.27E-08	0.0003358
18	32192058	rs7244352	*FHOD3*	-0.5103	-1.273	0.2044	0.2778	-2.496	-4.581	0.0126	4.85E-06
